# Cubic exact solutions for the estimation of pairwise haplotype frequencies: implications for linkage disequilibrium analyses and a web tool 'CubeX'

**DOI:** 10.1186/1471-2105-8-428

**Published:** 2007-11-02

**Authors:** Tom R Gaunt, Santiago Rodríguez, Ian NM Day

**Affiliations:** 1 Bristol Genetic Epidemology Laboratories (BGEL) and MRC Centre for Causal Analyses in Translational Epidemiology (CAiTE), Department of Social Medicine, University of Bristol, Canynge Hall, Whiteladies Road, Bristol, BS8 2PR, UK

## Abstract

**Background:**

The frequency of a haplotype comprising one allele at each of two loci can be expressed as a cubic equation (the 'Hill equation'), the solution of which gives that frequency. Most haplotype and linkage disequilibrium analysis programs use iteration-based algorithms which substitute an estimate of haplotype frequency into the equation, producing a new estimate which is repeatedly fed back into the equation until the values converge to a maximum likelihood estimate (expectation-maximisation).

**Results:**

We present a program, "CubeX", which calculates the biologically possible exact solution(s) and provides estimated haplotype frequencies, D', r^2 ^and *χ*^2 ^values for each. CubeX provides a "complete" analysis of haplotype frequencies and linkage disequilibrium for a pair of biallelic markers under situations where sampling variation and genotyping errors distort sample Hardy-Weinberg equilibrium, potentially causing more than one biologically possible solution. We also present an analysis of simulations and real data using the algebraically exact solution, which indicates that under perfect sample Hardy-Weinberg equilibrium there is only one biologically possible solution, but that under other conditions there may be more.

**Conclusion:**

Our analyses demonstrate that lower allele frequencies, lower sample numbers, population stratification and a possible |D'| value of 1 are particularly susceptible to distortion of sample Hardy-Weinberg equilibrium, which has significant implications for calculation of linkage disequilibrium in small sample sizes (eg HapMap) and rarer alleles (eg paucimorphisms, q < 0.05) that may have particular disease relevance and require improved approaches for meaningful evaluation.

## Background

Linkage disequilibrium (LD) describes the condition that occurs when alleles at different loci are non-randomly associated in a given population. Under LD the frequency (*f*_11_) of a haplotype (*h*_11_) representing the "1" allele at two loci is significantly more or less than the product of the respective allele frequencies. Characterisation of LD is important in medical genetics, influencing association mapping of trait loci and providing information on interactions between genes [1,2]. LD is the result of a shared history of mutation and recombination, and other factors including: genetic drift, population growth, admixture, population structure, the ages of the polymorphisms, the physical distance separating them and the effects of selective pressure [3].

For unrelated individuals the estimation of LD relies on the estimation of haplotype frequencies. In a 3 × 3 table for a biallelic marker the haplotype phase of all individuals is known with the exception of the centre cell (representing individuals heterozygous at both loci). The estimated frequency, f^11
 MathType@MTEF@5@5@+=feaafiart1ev1aaatCvAUfKttLearuWrP9MDH5MBPbIqV92AaeXatLxBI9gBaebbnrfifHhDYfgasaacPC6xNi=xH8viVGI8Gi=hEeeu0xXdbba9frFj0xb9qqpG0dXdb9aspeI8k8fiI+fsY=rqGqVepae9pg0db9vqaiVgFr0xfr=xfr=xc9adbaqaaeGacaGaaiaabeqaaeqabiWaaaGcbaGafmOzayMbaKaadaWgaaWcbaGaeGymaeJaeGymaedabeaaaaa@2F44@, of the haplotype *h*_11 _is described by a cubic equation of the form

af^113+bf^112+cf^11+d=0
 MathType@MTEF@5@5@+=feaafiart1ev1aaatCvAUfKttLearuWrP9MDH5MBPbIqV92AaeXatLxBI9gBaebbnrfifHhDYfgasaacPC6xNi=xI8qiVKYPFjYdHaVhbbf9v8qqaqFr0xc9vqFj0dXdbba91qpepeI8k8fiI+fsY=rqGqVepae9pg0db9vqaiVgFr0xfr=xfr=xc9adbaqaaeGacaGaaiaabeqaaeqabiWaaaGcbaGaemyyaeMafmOzayMbaKaadaqhaaWcbaGaeGymaeJaeGymaedabaGaeG4mamdaaOGaey4kaSIaemOyaiMafmOzayMbaKaadaqhaaWcbaGaeGymaeJaeGymaedabaGaeGOmaidaaOGaey4kaSIaem4yamMafmOzayMbaKaadaWgaaWcbaGaeGymaeJaeGymaedabeaakiabgUcaRiabdsgaKjabg2da9iabicdaWaaa@424C@

that is adapted from Hill's equation (4) [4] with the constants defined under Methods. With f^11
 MathType@MTEF@5@5@+=feaafiart1ev1aaatCvAUfKttLearuWrP9MDH5MBPbIqV92AaeXatLxBI9gBaebbnrfifHhDYfgasaacPC6xNi=xH8viVGI8Gi=hEeeu0xXdbba9frFj0xb9qqpG0dXdb9aspeI8k8fiI+fsY=rqGqVepae9pg0db9vqaiVgFr0xfr=xfr=xc9adbaqaaeGacaGaaiaabeqaaeqabiWaaaGcbaGafmOzayMbaKaadaWgaaWcbaGaeGymaeJaeGymaedabeaaaaa@2F44@ and the allele frequencies, all four haplotype frequencies can be calculated, thus estimating the unknown proportions of the middle cell.

Several approaches exist for solving equation (1), the solution of which enables estimation of haplotype frequencies and LD coefficients. The first approach uses iteration-based algorithms. An initial estimate of haplotype frequency (either random, or based on the known haplotype numbers) is substituted into the equation, providing a new estimate. This is then fed back into the equation and the expectation-maximisation (EM) process repeated until the values converge. This is the basis both of the algorithm described by Hill in 1974 for the estimation of pairwise haplotype frequencies [4], and of other EM algorithms that enable the estimation of multilocus haplotype frequencies. Many programs exist that utilise variations on this approach, including: GOLD [5], GOLDsurfer [6], MIDAS [7], Haploview [8] and many others reviewed in [9-12]. The potential problem for these approaches is that algorithms may converge on one of the alternative roots of the cubic equation (a local maximum rather than the global maximum).

Other approaches include parsimony, eg HAPAR [13] and Bayesian algorithms, eg PHASE [14-16]. Parsimony and Bayesian methods are both better suited to estimating individual haplotypes than EM approaches, while Bayesian and EM methods are useful for estimating population frequencies [11].

An alternative approach would be exact solution, such as *Cardan's solution *[17] of the generalized cubic equation (of which equation (1) is an example). This provides all roots to the cubic equation, from which we can select those that are both *real *(i.e. not a complex number) and *biologically possible*. If more than one solution exists then the likelihoods of the different solutions can be compared and an informed evaluation made of the result. Theoretically, the non-iterative approach may be computationally less intensive and more accurate, but computational efficiency and accuracy will be software and platform dependent.

## Implementation

Hill assumed random mating and Hardy Weinberg Equilibrium (HWE) [4]. Rearranging terms for consequent diplotype frequency expectations for two biallelic loci Luo and Suhai [18] obtained equation 1 given in the introduction (here redefining f^11
 MathType@MTEF@5@5@+=feaafiart1ev1aaatCvAUfKttLearuWrP9MDH5MBPbIqV92AaeXatLxBI9gBaebbnrfifHhDYfgasaacPC6xNi=xH8viVGI8Gi=hEeeu0xXdbba9frFj0xb9qqpG0dXdb9aspeI8k8fiI+fsY=rqGqVepae9pg0db9vqaiVgFr0xfr=xfr=xc9adbaqaaeGacaGaaiaabeqaaeqabiWaaaGcbaGafmOzayMbaKaadaWgaaWcbaGaeGymaeJaeGymaedabeaaaaa@2F44@ as *x*, *a3 *as *a*, *a2 *as *b*, *a1 *as *c *and *a *as *d *for convenience): *ax*^3 ^+ *bx*^2 ^+ *cx *+ *d *= 0, where *a *= 4*n*; *b *= 2*n *(1 - 2*p *- 2*q*) - 2(2*n*_11 _+ *n*_12 _+ *n*_21_) - *n*_22_; *c *= 2*npq *- (2*n*_11 _+ *n*_12 _+ *n*_21_)(1 - 2*p *- 2*q*) - *n*_22_(1 - *p *- *q*); *d *= -(2*n*_11 _+ *n*_12 _+ *n*_21_)*pq*; *n = *number of subjects; *p *= common allele freq of locus 1; *q *= common allele freq of locus 2; *n*_11 _is the number of subjects who are homozygous for the commoner allele at both loci; *n*_12 _are common homozygous at locus 1 and heterozygous at locus 2; *n*_21 _are heterozygous at locus 1 and common homozygous at locus 2; *n*_22 _are heterozygous at both loci [18]. Equation 1 can be solved exactly for *x *(with 1 to 3 real number solutions).

We have adopted the Nickalls treatment of the Cardan solution of the generalized cubic equation [17], and written a Python [19] program "CubeX" to solve equation 1 exactly. In CubeX, after calculation of constants *a*-*d *from diplotypic data the following are calculated:

*x*_*N *_= -*b*/(*3a*); *δ*^2 ^= *(b*^2 ^-*3ac)/9a*^2^; *h*^2 ^= 4*a*^2^*δ*^6^; *y*_*N *_= *ax*_*N*_^3 ^+ *bx*_*N*_^2 ^+ *cx*_*N *_+ *d*.

The discriminant Δ_3 _= *y*_*N*_^2 ^- *h*^2 ^is then used to determine the outcome in real roots (without having to go through complex number intermediates or ambiguities), with three possible outcomes:

Outcome 1: if *y*_*N*_^2 ^> *h*^2 ^there will be only one real root (*α*) given by

α=xN+12a(−yN+yN2−h2)3+12a(−yN−yN2−h2)3
 MathType@MTEF@5@5@+=feaafiart1ev1aaatCvAUfKttLearuWrP9MDH5MBPbIqV92AaeXatLxBI9gBaebbnrfifHhDYfgasaacPC6xNi=xI8qiVKYPFjYdHaVhbbf9v8qqaqFr0xc9vqFj0dXdbba91qpepeI8k8fiI+fsY=rqGqVepae9pg0db9vqaiVgFr0xfr=xfr=xc9adbaqaaeGacaGaaiaabeqaaeqabiWaaaGcbaacciGae8xSdeMaeyypa0JaemiEaG3aaSbaaSqaaiabd6eaobqabaGccqGHRaWkdaGcbaqaaKqbaoaalaaabaGaeGymaedabaGaeGOmaiJaemyyaegaaOWaaeWaaeaacqGHsislcqWG5bqEdaWgaaWcbaGaemOta4eabeaakiabgUcaRmaakaaabaGaemyEaK3aa0baaSqaaiabd6eaobqaaiabikdaYaaakiabgkHiTiabdIgaOnaaCaaaleqabaGaeGOmaidaaaqabaaakiaawIcacaGLPaaaaSqaaiabiodaZaaakiabgUcaRmaakeaabaqcfa4aaSaaaeaacqaIXaqmaeaacqaIYaGmcqWGHbqyaaGcdaqadaqaaiabgkHiTiabdMha5naaBaaaleaacqWGobGtaeqaaOGaeyOeI0YaaOaaaeaacqWG5bqEdaqhaaWcbaGaemOta4eabaGaeGOmaidaaOGaeyOeI0IaemiAaG2aaWbaaSqabeaacqaIYaGmaaaabeaaaOGaayjkaiaawMcaaaWcbaGaeG4mamdaaaaa@582E@

Outcome 2: if *y*_*N*_^2 ^= *h*^2 ^there are three real roots (*α*, *β *and *γ*) and *α *and *β *are equal. For a value of μ=yN2a3
 MathType@MTEF@5@5@+=feaafiart1ev1aaatCvAUfKttLearuWrP9MDH5MBPbIqV92AaeXatLxBI9gBaebbnrfifHhDYfgasaacPC6xNi=xH8viVGI8Gi=hEeeu0xXdbba9frFj0xb9qqpG0dXdb9aspeI8k8fiI+fsY=rqGqVepae9pg0db9vqaiVgFr0xfr=xfr=xc9adbaqaaeGacaGaaiaabeqaaeqabiWaaaGcbaacciGae8hVd0Maeyypa0ZaaOqaaeaajuaGdaWcaaqaaiabdMha5naaBaaabaGaemOta4eabeaaaeaacqaIYaGmcqWGHbqyaaaaleaacqaIZaWmaaaaaa@3541@:

*α *= *x*_*N *_+ *μ*

*β *= *x*_*N *_+ *μ*

*γ *= *x*_*N *_- 2*μ*

Outcome 3: if *y*_*N*_^2 ^<*h*^2 ^there are three real roots (*α*, *β *and *γ*). Where θ=arccos⁡(−yN/h)3
 MathType@MTEF@5@5@+=feaafiart1ev1aaatCvAUfKttLearuWrP9MDH5MBPbIqV92AaeXatLxBI9gBaebbnrfifHhDYfgasaacPC6xNi=xH8viVGI8Gi=hEeeu0xXdbba9frFj0xb9qqpG0dXdb9aspeI8k8fiI+fsY=rqGqVepae9pg0db9vqaiVgFr0xfr=xfr=xc9adbaqaaeGacaGaaiaabeqaaeqabiWaaaGcbaacciGae8hUdeNaeyypa0tcfa4aaSaaaeaacyGGHbqycqGGYbGCcqGGJbWycqGGJbWycqGGVbWBcqGGZbWCcqGGOaakcqGHsislcqWG5bqEdaWgaaqaaiabd6eaobqabaGaei4la8IaemiAaGMaeiykaKcabaGaeG4mamdaaaaa@3FEF@:

*α *= *x*_*N *_+ *2δ*cos*θ*

*β *= *x*_*N *_+ *2δ*cos(2*π*/3 + *θ*)

*γ *= *x*_*N *_+ *2δ*cos(4π/3 + *θ*)

Values for D' and r^2 ^are calculated as previously described [20,21]:

D=(f^11×f^22)−(f^12×f^21)
 MathType@MTEF@5@5@+=feaafiart1ev1aaatCvAUfKttLearuWrP9MDH5MBPbIqV92AaeXatLxBI9gBaebbnrfifHhDYfgasaacPC6xNi=xI8qiVKYPFjYdHaVhbbf9v8qqaqFr0xc9vqFj0dXdbba91qpepeI8k8fiI+fsY=rqGqVepae9pg0db9vqaiVgFr0xfr=xfr=xc9adbaqaaeGacaGaaiaabeqaaeqabiWaaaGcbaGaeeiraqKaeyypa0JaeiikaGIafmOzayMbaKaadaWgaaWcbaGaeGymaeJaeGymaedabeaakiabgEna0kqbdAgaMzaajaWaaSbaaSqaaiabikdaYiabikdaYaqabaGccqGGPaqkcqGHsislcqGGOaakcuWGMbGzgaqcamaaBaaaleaacqaIXaqmcqaIYaGmaeqaaOGaey41aqRafmOzayMbaKaadaWgaaWcbaGaeGOmaiJaeGymaedabeaakiabcMcaPaaa@44A9@

D_max _= min [*p*(1-*q*),(1-*p*)*q*] **if **D > 0 **or **D_max _= min [*pq*, (1-*p*)(1-*q*)] **if **D < 0

D' = D/D_max _

r^2 ^= D^2^/(*p*(1-*p*)*q*(1-*q*))

Diplotype frequencies based on the estimated haplotype frequencies are compared to the input diplotype frequencies by a *χ*^2 ^test, which effectively tests sample deviation from the null hypothesis of HWE for the diplotypes formed of the four haplotypes. The number of degrees of freedom is equal to the number of observations (diplotype counts) minus four estimated parameters which are either three haplotypes (the fourth can be inferred) and D, or one haplotype, two allele frequencies and D. If nine different diplotypes are observed the number of degrees of freedom is therefore five. For each empty cell in the 3 × 3 the number of degrees of freedom is reduced by one. If the user knows there are only three haplotypes present (and therefore six diplotypes) then there are only three estimated parameters (D is inferred by the three haplotype frequencies) and 3 df. It is important to note that in the latter case neither cubic solution nor iteration is necessary as the haplotype frequencies can be directly counted from the diplotype data. If the user believes that there are only three alleles and hence six diplotypes, but there are non-zero values for any of the other three possible diplotypes, then reconsideration of the technical veracity of the data and of the homogeneity of the population sample would be wise.

## Results

Solutions are considered biologically possible when f^11
 MathType@MTEF@5@5@+=feaafiart1ev1aaatCvAUfKttLearuWrP9MDH5MBPbIqV92AaeXatLxBI9gBaebbnrfifHhDYfgasaacPC6xNi=xH8viVGI8Gi=hEeeu0xXdbba9frFj0xb9qqpG0dXdb9aspeI8k8fiI+fsY=rqGqVepae9pg0db9vqaiVgFr0xfr=xfr=xc9adbaqaaeGacaGaaiaabeqaaeqabiWaaaGcbaGafmOzayMbaKaadaWgaaWcbaGaeGymaeJaeGymaedabeaaaaa@2F44@ and the derived f^12
 MathType@MTEF@5@5@+=feaafiart1ev1aaatCvAUfKttLearuWrP9MDH5MBPbIqV92AaeXatLxBI9gBaebbnrfifHhDYfgasaacPC6xNi=xH8viVGI8Gi=hEeeu0xXdbba9frFj0xb9qqpG0dXdb9aspeI8k8fiI+fsY=rqGqVepae9pg0db9vqaiVgFr0xfr=xfr=xc9adbaqaaeGacaGaaiaabeqaaeqabiWaaaGcbaGafmOzayMbaKaadaWgaaWcbaGaeGymaeJaeGOmaidabeaaaaa@2F46@, f^21
 MathType@MTEF@5@5@+=feaafiart1ev1aaatCvAUfKttLearuWrP9MDH5MBPbIqV92AaeXatLxBI9gBaebbnrfifHhDYfgasaacPC6xNi=xH8viVGI8Gi=hEeeu0xXdbba9frFj0xb9qqpG0dXdb9aspeI8k8fiI+fsY=rqGqVepae9pg0db9vqaiVgFr0xfr=xfr=xc9adbaqaaeGacaGaaiaabeqaaeqabiWaaaGcbaGafmOzayMbaKaadaWgaaWcbaGaeGOmaiJaeGymaedabeaaaaa@2F46@ and f^22
 MathType@MTEF@5@5@+=feaafiart1ev1aaatCvAUfKttLearuWrP9MDH5MBPbIqV92AaeXatLxBI9gBaebbnrfifHhDYfgasaacPC6xNi=xH8viVGI8Gi=hEeeu0xXdbba9frFj0xb9qqpG0dXdb9aspeI8k8fiI+fsY=rqGqVepae9pg0db9vqaiVgFr0xfr=xfr=xc9adbaqaaeGacaGaaiaabeqaaeqabiWaaaGcbaGafmOzayMbaKaadaWgaaWcbaGaeGOmaiJaeGOmaidabeaaaaa@2F48@ all fall within the range 0 to 1 (i.e. f^11,f^12,f^21,f^22∈[0,1]
 MathType@MTEF@5@5@+=feaafiart1ev1aaatCvAUfKttLearuWrP9MDH5MBPbIqV92AaeXatLxBI9gBaebbnrfifHhDYfgasaacPC6xNi=xH8viVGI8Gi=hEeeu0xXdbba9frFj0xb9qqpG0dXdb9aspeI8k8fiI+fsY=rqGqVepae9pg0db9vqaiVgFr0xfr=xfr=xc9adbaqaaeGacaGaaiaabeqaaeqabiWaaaGcbaGafmOzayMbaKaadaWgaaWcbaGaeGymaeJaeGymaedabeaakiabcYcaSiqbdAgaMzaajaWaaSbaaSqaaiabigdaXiabikdaYaqabaGccqGGSaalcuWGMbGzgaqcamaaBaaaleaacqaIYaGmcqaIXaqmaeqaaOGaeiilaWIafmOzayMbaKaadaWgaaWcbaGaeGOmaiJaeGOmaidabeaakiabgIGiolabcUfaBjabicdaWiabcYcaSiabigdaXiabc2faDbaa@4329@) and add up to 1. This constraint is tighter than those described elsewhere [22] as it relies on the inherent assumption of representative sampling and HWE, an extreme chance distortion of which could lead to three solutions at SNP allele frequencies of 0.5 in sample data drawn from a population (if all samples are heterozygous at both loci the following are possible: all could be diplotype 11/22, all could be diplotype 12/21, or there could be a combination of both).

### Number of solutions to the cubic equation with simulated data

**Figure 1:**
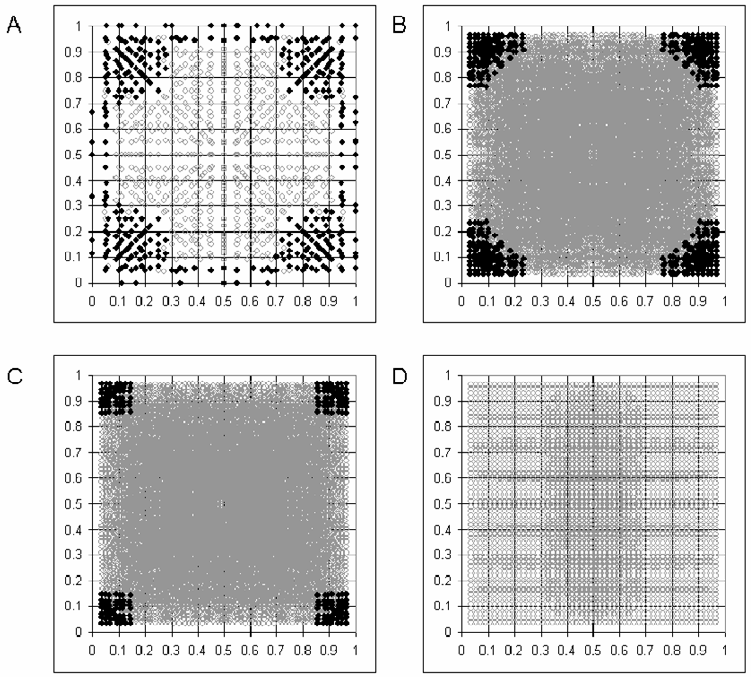
**Simulated data in which HWE is observed to the limit of rounding errors (whole number values for counts of individuals)**. (A) Number of biologically possible solutions to the cubic equation in (A) 10 individuals; (B) 60 individuals; (C) 100 individuals (D) 1000 individuals. x-axis: allele frequency of SNP1, y-axis: allele frequency of SNP2. Black = more than one solution. Grey = one solution.

We have calculated the number of possible solutions to the cubic equation for genotypes of simulated pairs of SNPs with a range of allele frequencies for a range of sample sizes. The genotype numbers were calculated assuming HWE with a wide range of LD situations for the two SNPs. This was achieved by simulating all combinations of haplotype frequencies between 0 and 1, at intervals of 1/55, that add up to 1. These haplotype frequencies were then converted to diplotype frequencies according to Hardy-Weinberg equilibrium. The results are plotted in Figure 1. Small samples result in minor deviations from sample HWE, allowing more than one solution. The smaller the sample size, the greater the range of allele frequencies over which this occurs. A sample of 10 subjects allows more than one biologically possible solution at a wide range of allele frequencies (Figure 1A). With 60 individuals a broad range of allele frequencies is still affected (Figure 1B) – this has implications for analyses based on the HapMap CEU dataset of 60 unrelated individuals [23,24]. At 100 individuals (Figure 1C) the problem is limited to allele frequencies below 15% (Figure 1C), while the plot for 1000 individuals shows no condition under which there is more than one biologically possible solution (Figure 1D). This last observation is because under perfect sample HWE (infinite samples) the number of *biologically possible *solutions is always 1, despite the number of *real *solutions exceeding 1 at lower allele frequencies (data not shown).

### Number of solutions to the cubic equation with real data

**Figure 2:**
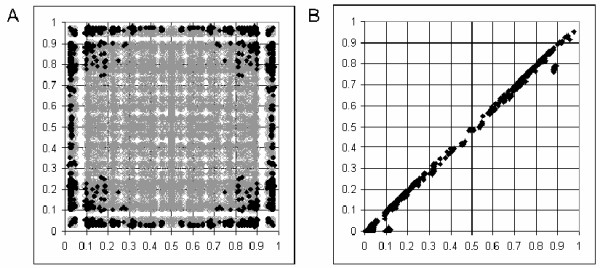
**Evaluation of number of solutions for real data**. (A) Number of biologically possible solutions over a range of allele frequencies using a large sample of SNP data (Chr. 17:60 to 60.5 MB, 121 SNPs) from the HapMap project [23,24]. x-axis: allele frequency of SNP1, y-axis: allele frequency of SNP2. Black = more than one solution. Grey = one solution. (B) Comparison of two solutions within the dataset. x-axis: higher value solution, y-axis: lower value solution.

**Figure 3:**
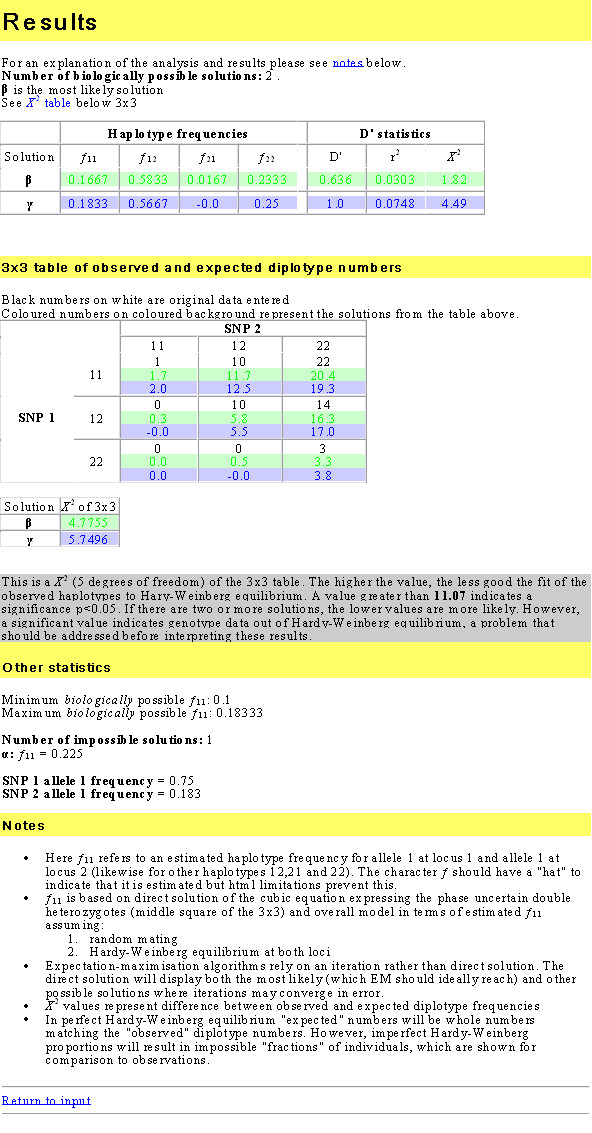
**Screenshot of results screen from CubeX online analysis program**. In this example there are two biologically possible solutions. Results for both are shown (upper table), and observed (input values) and expected diplotype frequencies (for the two solutions) displayed for comparison (lower table).

**Figure 4:**
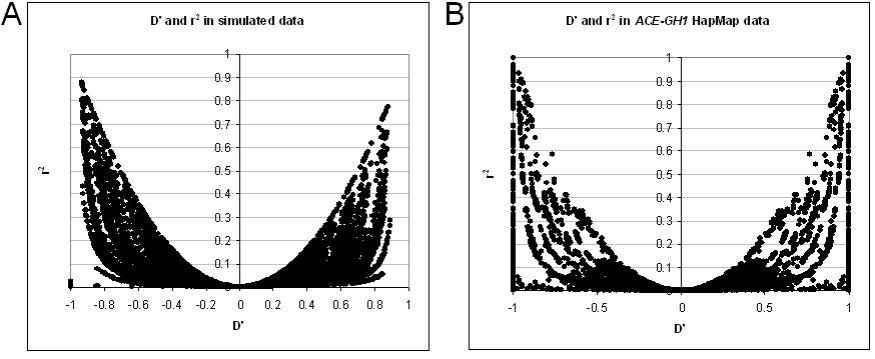
The range of LD in datasets using the CubeX tool to calculate r2 and D'. (A) Simulated data. D' on x-axis, r^2 ^on y axis. (B) Real SNP data (Chr. 17:60 to 60.5 MB, 121 SNPs) from the HapMap project [23,24]. D' on x-axis, r^2 ^on y axis.

We have also calculated the number of solutions to equation 1 for a set of real data from the HapMap project [23,24]. These were a selection of SNPs from the *ACE*-*GH1 *region of chromosome 17 for the CEU population (60 unrelated individuals). Figure 2A shows that at the lower allele frequencies the possibility of more than one real solution to the cubic equation begins to arise. This is consistent with the simulated data for 60 samples (Figure 1B), except that a broader range of allele frequencies is affected. This is probably due to the inherent errors of real data increasing the deviations from HWE relative to near-perfect simulated data. In most cases of multiple solutions only two of the three real roots are biologically possible. Figure 2B compares these two values, indicating that in most cases the differences in estimated haplotype are small. In the minority of cases with three solutions these fit the same pattern. However, this can have major consequences for the calculation of D' (as illustrated in Figure 3). Note that D' and r^2 ^behave quite differently in this respect, and r^2 ^is much less affected. However, as a |D'| of 1 indicates the existence of three or less haplotypes (r^2 ^of 1 indicates two haplotypes), |D'| is a good indicator of haplotype block structure, with a value of exactly 1 suggesting little or no recombination between two loci, and a value less than 1 supporting a break-down of LD. In fact CubeX provides both D' and r^2^, allowing the user to select their measure of preference. Figure 4 illustrates the relationship between these two measures in the simulated and real datasets, which clarifies how a large |D'| value can be observed with a low r^2 ^value, but the key point is that a |D'| of 1 indicates complete LD (i.e. three or less haplotypes) despite a low r^2^.

### Comparison of the cubic exact solution with other approaches

For the purposes of comparison we have analysed two datasets with PHASE [16], MIDAS [7] (Hill EM) and CubeX. The first is a dataset of directly haplotyped samples comprising 80 subjects from 3 ethnic groups (Asian, African and Caucasian) for *APOE *[25]. Although all but one SNP was in Hardy-Weinberg equilibrium, this dataset has the potential to invalidate some of the assumptions of the programs due to the mixture of ethnicity. However, this provides a useful substrate on which to test the influence of stratification on the outcome of the cubic exact solution. The second dataset is a set of multi-locus phased data from HapMap CEU samples [23,24] for the *IGF2 *gene region. Although these have not been directly haplotyped, the multi-locus phased haplotypes are expected to be very accurate, and this dataset comprises Caucasians, so will not suffer from the same stratification issues. We tested the programs on pair-wise subsets of these data.

For the *APOE *[25] dataset the data are presented in Additional File 1, with a selected summary in Table 1. The subset in Table 1 demonstrate the advantage of being provided with all possible solutions by CubeX, but also demonstrates that all three approaches can be wrong. To summarise the outcome, PHASE [16] and MIDAS [7] (Hill EM) both matched the real counts in 28 of 36 SNP pairs, while CubeX matched real counts in 33 of 36 SNP pairs (for one of its solutions). However, in five of those cases the user would need to determine which of the two CubeX solutions to use based on their prior knowledge of the LD structure in the region (i.e. do they expect three or four haplotypes). This comparison confirms the risk of EM finding a local maximum when there is more than one biologically possible solution, and suggests that CubeX may offer advantages in stratified datasets or datasets with low SNP minor allele frequencies (confirming the results from simulated data above).

**Table 1:** Illustrative examples of comparison of CubeX with PHASE [16] and MIDAS [7] (Hill EM).

			**Haplotype frequencies (rounded to 5 decimal places)**						**Haplotype numbers (rounded to nearest haplotype)**					
**Example**	**SNP pair**	**Haplotype**	**REAL frequency**	**PHASE frequency**	**MIDAS frequency**	**CUBEX alpha**	**CUBEX beta**	**CUBEX gamma**	**REAL number**	**PHASE number**	**MIDAS number**	**CUBEX alpha**	**CUBEX beta**	**CUBEX gamma**

1	Pair1_2	AC	0.0875	0.08689	0.0875	na	0.0875	na	**14**	14	14	na	14	na
	Pair1_2	AT	0.725	0.72561	0.725	na	0.725	na	**116**	116	116	na	116	na
	Pair1_2	TC	0	0.00061	0	na	0	na	**0**	0	0	na	0	na
	Pair1_2	TT	0.1875	0.18689	0.1875	na	0.1875	na	**30**	30	30	na	30	na
														
2	Pair1_5	AG	0.75	0.75318	0.75478	0.75478	na	0.75	**120**	121 *	121 *	121 *	na	120
	Pair1_5	AA	0.0625	0.05932	0.05772	0.05772	na	0.0625	**10**	9 *	9 *	9 *	na	10
	Pair1_5	TG	0.1875	0.18432	0.18272	0.18272	na	0.1875	**30**	29 *	29 *	29 *	na	30
	Pair1_5	TA	0	0.00318	0.00478	0.00478	na	0	**0**	1 *	1 *	1 *	na	0
														
3	Pair1_9	AT	0.05625	0.06477	0.05633	na	0.0563	0.075	**9**	10 *	9	na	9	12 *
	Pair1_9	AC	0.75625	0.74773	0.75617	na	0.7562	0.7375	**121**	120 *	121	na	121	118 *
	Pair1_9	TT	0.01875	0.01023	0.01867	na	0.0187	0	**3**	2 *	3	na	3	0 *
	Pair1_9	TC	0.16875	0.17727	0.16883	na	0.1688	0.1875	**27**	28 *	27	na	27	30 *
														
4	Pair2_3	CG	0.05625	0.04724	0.0465	0.0465	na	na	**9**	8 *	7 *	7 *	na	na
	Pair2_3	CT	0.03125	0.04026	0.041	0.041	na	na	**5**	6 *	7 *	7 *	na	na
	Pair2_3	TG	0.48125	0.49026	0.491	0.491	na	na	**77**	78 *	79 *	79 *	na	na
	Pair2_3	TT	0.43125	0.42224	0.4215	0.4215	na	na	**69**	68 *	67 *	67 *	na	na
														
5	pair5_9	GT	0.075	0.07313	0.06664	na	0.0666	0.075	**12**	12	11 *	na	11 *	12
	pair5_9	GC	0.8625	0.86437	0.87086	na	0.8709	0.8625	**138**	138	139 *	na	139 *	138
	pair5_9	AT	0	0.00187	0.00836	na	0.0084	0	**0**	0	1 *	na	1 *	0
	pair5_9	AC	0.0625	0.06063	0.05414	na	0.0541	0.0625	**10**	10	9 *	na	9 *	10

A selection of comparisons using direct haplotyped APOE data [25]. Full data are present as a additional table. For haplotype numbers (rounded to the nearest number) incorrect answers are marked '*', correct answers are unmarked. Examples: (1) Phase, MIDAS and CubeX (1 solution) give correct answer. (2) Only CubeX gives the correct answer as one of its two solutions. (3) MIDAS and CubeX give the correct answer, PHASE and the other CubeX solution are wrong. (4) All three approaches are wrong. (5) PHASE and CubeX give the correct answer, MIDAS and the other CubeX solution are wrong.

For the HapMap [23,24]* IGF2 *region data (comprising SNPs rs3802971, rs734351, rs3213221, rs4244808, rs1003483, rs3741208, rs1004446, rs4320932 and rs7924316) CubeX gives only one solution in all cases, and there is little difference between the outcome of the three approaches (Additional File 2). This confirms that in situations of higher allele frequencies there is less of an issue with multiple biologically possible solutions to the cubic equation, and iterative approaches are completely acceptable.

## Discussion

We have written an online program, "CubeX", to enable simple analysis of the biologically possible estimated haplotypes for pairs of biallelic markers. This program takes data from a pair of markers as a standard 3 × 3 table of nine diplotypes, generates cubic exact solutions to equation 1 and generates output in the format shown in Figure 3. The number of possible solutions is shown, followed by haplotype frequencies and LD statistics for those solutions. Below that a duplicate of the 3 × 3 input table is displayed with the addition of expected absolute diplotype frequencies calculated from the haplotype frequencies. The difference between these and the input data are subjected to a *χ*^2 ^test, which effectively tests sample deviation from the null hypothesis of HWE for the diplotypes formed of the four haplotypes. However, the interpretation of solutions depends on the prior hypothesis. In the example in Figure 3, although solution *γ *exhibits a slightly worse *χ*^2 ^fit than solution *β*, the former is consistent with a prior hypothesis of only three of the four haplotypes existing (see Figure 5 in reference [7]), which is biologically likely in the absence of recombination between any two loci. In fact, in all tested cases in Figure 2 generating more than one solution, the diplotype data included zero values in at least one corner cell and the two adjacent edge cells of the 3 × 3 (i.e. where one possible solution has a |D'| = 1, although it should be noted that more than one solution can occur without zero values if double heterozygotes are greatly over-represented). This suggests that the principal issue is whether three or four haplotypes exist, and in these cases the prior hypothesis (based on distance and recombination rates) is of utmost importance. If input data for individual SNPs are significantly out of HWE a warning message is given at the top of the page. For completeness, the biologically impossible real number solutions are displayed at the bottom, along with minimum and maximum biologically possible values for f^11
 MathType@MTEF@5@5@+=feaafiart1ev1aaatCvAUfKttLearuWrP9MDH5MBPbIqV92AaeXatLxBI9gBaebbnrfifHhDYfgasaacPC6xNi=xH8viVGI8Gi=hEeeu0xXdbba9frFj0xb9qqpG0dXdb9aspeI8k8fiI+fsY=rqGqVepae9pg0db9vqaiVgFr0xfr=xfr=xc9adbaqaaeGacaGaaiaabeqaaeqabiWaaaGcbaGafmOzayMbaKaadaWgaaWcbaGaeGymaeJaeGymaedabeaaaaa@2F44@ and allele frequencies. This program provides a convenient utility for researchers to both analyse data for haplotype frequencies and LD statistics and to check previous analyses for potential problems caused by multiple solutions.

Under perfect sample HWE the frequencies of all haplotypes can be directly inferred from the corresponding corner diplotypes of the 3 × 3. For example: n11=n f^112
 MathType@MTEF@5@5@+=feaafiart1ev1aaatCvAUfKttLearuWrP9MDH5MBPbIqV92AaeXatLxBI9gBaebbnrfifHhDYfgasaacPC6xNi=xH8viVGI8Gi=hEeeu0xXdbba9frFj0xb9qqpG0dXdb9aspeI8k8fiI+fsY=rqGqVepae9pg0db9vqaiVgFr0xfr=xfr=xc9adbaqaaeGacaGaaiaabeqaaeqabiWaaaGcbaGaemOBa42aaSbaaSqaaiabigdaXiabigdaXaqabaGccqGH9aqpcqqGUbGBcqqGGaaicuWGMbGzgaqcamaaDaaaleaacqaIXaqmcqaIXaqmaeaacqaIYaGmaaaaaa@36E2@, so f^11=n11n
 MathType@MTEF@5@5@+=feaafiart1ev1aaatCvAUfKttLearuWrP9MDH5MBPbIqV92AaeXatLxBI9gBaebbnrfifHhDYfgasaacPC6xNi=xH8viVGI8Gi=hEeeu0xXdbba9frFj0xb9qqpG0dXdb9aspeI8k8fiI+fsY=rqGqVepae9pg0db9vqaiVgFr0xfr=xfr=xc9adbaqaaeGacaGaaiaabeqaaeqabiWaaaGcbaGafmOzayMbaKaadaWgaaWcbaGaeGymaeJaeGymaedabeaakiabg2da9maakaaabaqcfa4aaSaaaeaacqWGUbGBdaWgaaqaaiabigdaXiabigdaXaqabaaabaGaemOBa4gaaaWcbeaaaaa@35D8@. That being the case there are only two possible values for f^11
 MathType@MTEF@5@5@+=feaafiart1ev1aaatCvAUfKttLearuWrP9MDH5MBPbIqV92AaeXatLxBI9gBaebbnrfifHhDYfgasaacPC6xNi=xH8viVGI8Gi=hEeeu0xXdbba9frFj0xb9qqpG0dXdb9aspeI8k8fiI+fsY=rqGqVepae9pg0db9vqaiVgFr0xfr=xfr=xc9adbaqaaeGacaGaaiaabeqaaeqabiWaaaGcbaGafmOzayMbaKaadaWgaaWcbaGaeGymaeJaeGymaedabeaaaaa@2F44@, one positive and one negative, the latter being biologically impossible. Perfect sample HWE therefore results in only a single biologically possible solution to the cubic equation. In the case of extreme sample HWD where all samples fall within the middle cell of the 3 × 3, f^11
 MathType@MTEF@5@5@+=feaafiart1ev1aaatCvAUfKttLearuWrP9MDH5MBPbIqV92AaeXatLxBI9gBaebbnrfifHhDYfgasaacPC6xNi=xH8viVGI8Gi=hEeeu0xXdbba9frFj0xb9qqpG0dXdb9aspeI8k8fiI+fsY=rqGqVepae9pg0db9vqaiVgFr0xfr=xfr=xc9adbaqaaeGacaGaaiaabeqaaeqabiWaaaGcbaGafmOzayMbaKaadaWgaaWcbaGaeGymaeJaeGymaedabeaaaaa@2F44@ can contribute either a half, a quarter or none of the haplotypes to the middle cell. There are therefore three biologically possible solutions under conditions of extreme sample HWD. The results from real data confirm that in some cases more than one biologically possible solution to the cubic equation for haplotype frequency can exist. The simulations suggest that this occurs where small sample size, sampling errors or non-random mating result in a distortion of sample HWE, and demonstrates the importance of testing HWE before haplotype analyses. The greater the distortion of sample HWE the higher the allele frequency at which more than one solution can occur (hence, as described above, three solutions can occur at allele frequencies of 0.5 if all samples are heterozygous at both loci). In these cases the cubic exact algorithm gives all possible solutions and a test of HWE, while an iteration-based method would only give one. This supports the hypothesis that the cubic exact approach is superior to iteration-based methods in real-world datasets where sample data rarely fit exactly to HWE (note that sample may differ from population in HWE statistics – here we refer to sample HWE). This is particularly important in the analysis of low frequency SNPs and paucimorphisms [26-28], for which different solutions can significantly distort D' results, despite the relatively similar solutions giving similar r^2 ^results. In all the observed data with two solutions there were no occasions in which r^2 ^exceeded 0.3 for any biologically possible solution, and in most cases there is only a small difference in r^2 ^between biologically possible solutions. The largest effect is on D'. On the basis of empirical data and using different approaches to inference Wong *et al *showed that coding SNPs with minor allele frequencies <0.06 are likely to be of functional importance [29], and rarer alleles, haplotypes and diplotypes of causal importance have emerged in numerous disease contexts (eg. inflammatory bowel disease, hemochromatosis). In addition to being applicable and giving exact evaluation for D' analysis of common SNPs, the cubic exact solution may prove of particular value for evaluating "post-HapMap" and "post-dbSNP" rarer haplotypes, for fully evaluating D' estimates from datasets with greater deviations from the random mating and HWE assumptions and for fully evaluating LD in small datasets.

Finally, we have demonstrated by comparison with PHASE [16] and MIDAS [7] (Hill EM) that in certain situations (low minor allele frequency, population stratification) the cubic exact approach can perform better for pair-wise analyses than alternative approaches by indicating the existence of multiple solutions. However, our findings confirm that in most other situations iterative approaches are robust and accurate.

## Conclusion

We present a comprehensive analysis of the consequences of different variables on the number of solutions to the cubic equation for haplotype frequency. Our analyses demonstrate that lower allele frequencies, lower sample numbers and a possible |D'| value of 1 can result in more than one solution. This has significant implications for the calculation of LD in small sample sizes and with rarer alleles that may have particular disease relevance. This evaluation provides essential information for an understanding of the limitations of LD estimation, which is particularly relevant for genome-wide analyses (where sample sizes and allele frequencies can be low). Finally, we present a program "CubeX", freely available as an online program, which provides each of the biologically possible cubic exact solution(s) to equation 1 for haplotype frequency, enabling the user to identify the solution that best fits their prior hypothesis for number of haplotypes.

## Availability and Requirements

Project name: CubeX

Project home page: 

Operating system(s): Platform independent (web-based)

Programming language: Python 

Licence: CubeX licence available from 

Any restrictions to use by non-academics: royalty-free use allowed within terms of licence

## Abbreviations

EM – Expectation-Maximisation

HWE – Hardy-Weinberg Equilibrium

LD – Linkage Disequilibrium

## Authors' contributions

TRG wrote the CubeX program, ran the simulations and analyses and drafted the manuscript. SR advised on LD calculation and output format, tested the program and contributed to the manuscript. INMD drafted the solution to the cubic equation, advised on methods, tested the program and contributed to the manuscript. All authors read and approved the final manuscript.

## Supplementary Material

Additional file 1 Comparisons of PHASE, MIDAS and CubeX on *APOE *data (from [25]). A comparison of PHASE, MIDAS and CubeX for pairwise analysis of genotype data derived from directly observed multi-locus haplotypes. Click here for file

Additional file 2 Comparisons of PHASE, MIDAS and CubeX on HapMap *IGF2 *region data (from , [23,24]). A comparison of PHASE, MIDAS and CubeX for pairwise analysis of genotype data derived from statistically inferred long-range multi-locus haplotypes. Click here for file
