# Supplementary material for: Cubic exact solutions for the estimation of pairwise haplotype frequencies: implications for linkage disequilibrium analyses and a web tool 'CubeX'
Source: BMC Bioinformatics. 2007 Nov 2;8:428. doi: 10.1186/1471-2105-8-428 (PMC2180187; doi:10.1186/1471-2105-8-428)
Supplement: Additional file 1 — Comparisons of PHASE, MIDAS and CubeX on APOE data (from [25]). A comparison of PHASE, MIDAS and CubeX for pairwise analysis of genotype data derived from directly observed multi-locus haplotypes. [file 1471-2105-8-428-S1.pdf]

Supplementary File 1: Comparisons of PHASE, MIDAS and CubeX (CubeX-a, CubeX-b and CubeX-g for three solutions, na where a solution is not biologically possible) on *APOE* data (Orzack et al, Genetics 2003, 165: 915-928).

		Frequencies						Counts					
Pair	Haplotype	Real	PHASE	MIDAS	CubeX-a	CubeX-b	CubeX-g	Real	PHASE	MIDAS	CubeX-a	CubeX-b	CubeX-g
pair1_2	AC	0.08750	0.086891	0.08750	na	0.08750	na	14	14	14	na	14	na
pair1_2	AT	0.72500	0.725609	0.72500	na	0.72500	na	116	116	116	na	116	na
pair1_2	TC	0.00000	0.000609	0.00000	na	0.00000	na	0	0	0	na	0	na
pair1_2	TT	0.18750	0.186891	0.18750	na	0.18750	na	30	30	30	na	30	na
pair1_3	AG	0.43125	0.408210	0.40150	0.40150	na	na	69	65	64	64	na	na
pair1_3	AT	0.38125	0.404290	0.41100	0.41100	na	na	61	65	66	66	na	na
pair1_3	TG	0.10625	0.129290	0.13600	0.13600	na	na	17	21	22	22	na	na
pair1_3	TT	0.08125	0.058210	0.05150	0.05150	na	na	13	9	8	8	na	na
pair1_4	AG	0.50000	0.462193	0.45633	0.45633	na	na	80	74	73	73	na	na
pair1_4	AC	0.31250	0.350307	0.35617	0.35617	na	na	50	56	57	57	na	na
pair1_4	TG	0.13125	0.169057	0.17492	0.17492	na	na	21	27	28	28	na	na
pair1_4	TC	0.05625	0.018443	0.01258	0.01258	na	na	9	3	2	2	na	na
pair1_5	AG	0.75000	0.753177	0.75478	0.75478	na	0.75000	120	121	121	121	na	120
pair1_5	AA	0.06250	0.059323	0.05772	0.05772	na	0.06250	10	9	9	9	na	10
pair1_5	TG	0.18750	0.184323	0.18272	0.18272	na	0.18750	30	29	29	29	na	30
pair1_5	TA	0.00000	0.003177	0.00478	0.00478	na	0.00000	0	1	1	1	na	0

pair1_6	AG	0.80625	0.806250	0.80625	0.80625	na	na	129	129	129	129	na	na
pair1_6	AA	0.00625	0.006250	0.00625	0.00625	na	na	1	1	1	1	na	na
pair1_6	TG	0.18750	0.187500	0.18750	0.18750	na	na	30	30	30	30	na	na
pair1_6	TA	0.00000	0.00000	0.00000	0.00000	na	na	0	0	0	0	na	na
pair1_7	AT	0.73125	0.743570	0.74882	0.74882	na	na	117	119	120	120	na	na
pair1_7	AC	0.08125	0.068930	0.06368	0.06368	na	na	13	11	10	10	na	na
pair1_7	TT	0.15000	0.137680	0.13243	0.13243	na	na	24	22	21	21	na	na
pair1_7	TC	0.03750	0.049820	0.05507	0.05507	na	na	6	8	9	9	na	na
pair1_8	AC	0.79375	0.794742	0.79622	0.79622	na	0.79375	127	127	127	127	na	127
pair1_8	AT	0.01875	0.017758	0.01628	0.01628	na	0.01875	3	3	3	3	na	3
pair1_8	TC	0.18750	0.186508	0.18503	0.18503	na	0.18750	30	30	30	30	na	30
pair1_8	TT	0.00000	0.000992	0.00247	0.00247	na	0.00000	0	0	0	0	na	0
pair1_9	AT	0.05625	0.064770	0.05633	na	0.05633	0.07500	9	10	9	na	9	12
pair1_9	AC	0.75625	0.747730	0.75617	na	0.75617	0.73750	121	120	121	na	121	118
pair1_9	TT	0.01875	0.010230	0.01867	na	0.01867	0.00000	3	2	3	na	3	0
pair1_9	TC	0.16875	0.177270	0.16883	na	0.16883	0.18750	27	28	27	na	27	30
pair2_3	CG	0.05625	0.047239	0.04650	0.04650	na	na	9	8	7	7	na	na
pair2_3	CT	0.03125	0.040261	0.04100	0.04100	na	na	5	6	7	7	na	na
pair2_3	TG	0.48125	0.490261	0.49100	0.49100	na	na	77	78	79	79	na	na
pair2_3	TT	0.43125	0.422239	0.42150	0.42150	na	na	69	68	67	67	na	na

pair2_4	CG	0.05625	0.047710	0.04156	0.04156	na	na	9	8	7	7	na	na
pair2_4	CC	0.03125	0.039790	0.04594	0.04594	na	na	5	6	7	7	na	na
pair2_4	TG	0.57500	0.583540	0.58969	0.58969	na	na	92	93	94	94	na	na
pair2_4	TC	0.33750	0.328960	0.32281	0.32281	na	na	54	53	52	52	na	na
pair2_5	CG	0.08750	0.086881	0.08750	na	0.08750	na	14	14	14	na	14	na
pair2_5	CA	0.00000	0.000619	0.00000	na	0.00000	na	0	0	0	na	0	na
pair2_5	TG	0.85000	0.850619	0.85000	na	0.85000	na	136	136	136	na	136	na
pair2_5	TA	0.06250	0.061881	0.06250	na	0.06250	na	10	10	10	na	10	na
pair2_6	CG	0.08750	0.087500	0.08750	na	0.08750	na	14	14	14	na	14	na
pair2_6	TG	0.90625	0.906250	0.90625	na	0.90625	na	145	145	145	na	145	na
pair2_6	TA	0.00625	0.006250	0.00625	na	0.00625	na	1	1	1	na	1	na
pair2_6	CA	0.00000	0.00000	0.00000	na	0.00000	na	0	0	0	na	0	na
pair2_7	CT	0.08750	0.087158	0.08750	na	0.08750	na	14	14	14	na	14	na
pair2_7	CC	0.00000	0.000342	0.00000	na	0.00000	na	0	0	0	na	0	na
pair2_7	TT	0.79375	0.794092	0.79375	na	0.79375	na	127	127	127	na	127	na
pair2_7	TC	0.11875	0.118408	0.11875	na	0.11875	na	19	19	19	na	19	na
pair2_8	CC	0.08750	0.087500	0.08750	na	0.08750	na	14	14	14	na	14	na
pair2_8	TC	0.89375	0.893750	0.89375	na	0.89375	na	143	143	143	na	143	na
pair2_8	TT	0.01875	0.018750	0.01875	na	0.01875	na	3	3	3	na	3	na

pair2_8	CT	0.00000	0.00000	0.00000	na	0.00000	na	0	0	0	na	0	na
pair2_9	CT	0.05625	0.055341	0.05572	0.05572	na	na	9	9	9	9	na	na
pair2_9	CC	0.03125	0.032159	0.03178	0.03178	na	na	5	5	5	5	na	na
pair2_9	TT	0.01875	0.019659	0.01928	0.01928	na	na	3	3	3	3	na	na
pair2_9	TC	0.89375	0.892841	0.89322	0.89322	na	na	143	143	143	143	na	na
pair3_4	GG	0.51875	0.515764	0.51633	0.51633	na	na	83	83	83	83	na	na
pair3_4	GC	0.01875	0.021736	0.02117	0.02117	na	na	3	3	3	3	na	na
pair3_4	TG	0.11250	0.115486	0.11492	0.11492	na	na	18	18	18	18	na	na
pair3_4	TC	0.35000	0.347014	0.34758	0.34758	na	na	56	56	56	56	na	na
pair3_5	GG	0.53750	0.535869	0.53750	0.53750	na	na	86	86	86	86	na	na
pair3_5	GA	0.00000	0.001631	0.00000	0.00000	na	na	0	0	0	0	na	na
pair3_5	TG	0.40000	0.401631	0.40000	0.40000	na	na	64	64	64	64	na	na
pair3_5	TA	0.06250	0.060869	0.06250	0.06250	na	na	10	10	10	10	na	na
pair3_6	GG	0.53750	0.537500	0.53750	0.53750	na	na	86	86	86	86	na	na
pair3_6	TG	0.45625	0.456250	0.45625	0.45625	na	na	73	73	73	73	na	na
pair3_6	TA	0.00625	0.006250	0.00625	0.00625	na	na	1	1	1	1	na	na
pair3_6	GA	0.00000	0.00000	0.00000	0.00000	na	na	0	0	0	0	na	na
pair3_7	GT	0.51875	0.514617	0.51831	0.51831	na	na	83	82	83	83	na	na
pair3_7	GC	0.01875	0.022883	0.01919	0.01919	na	na	3	4	3	3	na	na

pair3_7	TT	0.36250	0.366633	0.36294	0.36294	na	na	58	59	58	58	na	na
pair3_7	TC	0.10000	0.095867	0.09956	0.09956	na	na	16	15	16	16	na	na
pair3_8	GC	0.51875	0.521530	0.51875	0.51875	na	na	83	83	83	83	na	na
pair3_8	GT	0.01875	0.015970	0.01875	0.01875	na	na	3	3	3	3	na	na
pair3_8	TC	0.46250	0.459720	0.46250	0.46250	na	na	74	74	74	74	na	na
pair3_8	TT	0.00000	0.002780	0.00000	0.00000	na	na	0	0	0	0	na	na
pair3_9	GT	0.07500	0.073183	0.07500	0.07500	na	na	12	12	12	12	na	na
pair3_9	GC	0.46250	0.464317	0.46250	0.46250	na	na	74	74	74	74	na	na
pair3_9	TT	0.00000	0.001817	0.00000	0.00000	na	na	0	0	0	0	na	na
pair3_9	TC	0.46250	0.460683	0.46250	0.46250	na	na	74	74	74	74	na	na
pair4_5	GG	0.56875	0.569979	0.56875	0.56875	na	na	91	91	91	91	na	na
pair4_5	GA	0.06250	0.061271	0.06250	0.06250	na	na	10	10	10	10	na	na
pair4_5	CG	0.36875	0.367521	0.36875	0.36875	na	na	59	59	59	59	na	na
pair4_5	CA	0.00000	0.001229	0.00000	0.00000	na	na	0	0	0	0	na	na
pair4_6	GG	0.63125	0.631250	0.63125	0.63125	na	na	101	101	101	101	na	na
pair4_6	CG	0.36250	0.362500	0.36250	0.36250	na	na	58	58	58	58	na	na
pair4_6	CA	0.00625	0.006250	0.00625	0.00625	na	na	1	1	1	1	na	na
pair4_6	GA	0.00000	0.00000	0.00000	0.00000	na	na	0	0	0	0	na	na
pair4_7	GT	0.51250	0.514330	0.51250	0.51250	na	na	82	82	82	82	na	na

pair4_7	GC	0.11875	0.116920	0.11875	0.11875	na	na	19	19	19	19	na	na
pair4_7	CT	0.36875	0.366920	0.36875	0.36875	na	na	59	59	59	59	na	na
pair4_7	CC	0.00000	0.001830	0.00000	0.00000	na	na	0	0	0	0	na	na
pair4_8	GC	0.61250	0.614713	0.61588	0.61588	na	0.61250	98	98	99	99	na	98
pair4_8	GT	0.01875	0.016537	0.01537	0.01537	na	0.01875	3	3	2	2	na	3
pair4_8	CC	0.36875	0.366537	0.36537	0.36537	na	0.36875	59	59	58	58	na	59
pair4_8	CT	0.00000	0.002213	0.00338	0.00338	na	0.00000	0	0	1	1	na	0
pair4_9	GT	0.07500	0.072439	0.07500	0.07500	na	na	12	12	12	12	na	na
pair4_9	GC	0.55625	0.558811	0.55625	0.55625	na	na	89	89	89	89	na	na
pair4_9	CT	0.00000	0.002561	0.00000	0.00000	na	na	0	0	0	0	na	na
pair4_9	CC	0.36875	0.366189	0.36875	0.36875	na	na	59	59	59	59	na	na
pair5_6	GG	0.93125	0.931250	0.93125	0.93125	na	na	149	149	149	149	na	na
pair5_6	GA	0.00625	0.006250	0.00625	0.00625	na	na	1	1	1	1	na	na
pair5_6	AG	0.06250	0.062500	0.06250	0.06250	na	na	10	10	10	10	na	na
pair5_6	AA	0.00000	0.00000	0.00000	0.00000	na	na	0	0	0	0	na	na
pair5_7	GT	0.88125	0.881114	0.88125	0.88125	na	0.81875	141	141	141	141	na	131
pair5_7	GC	0.05625	0.056386	0.05625	0.05625	na	0.11875	9	9	9	9	na	19
pair5_7	AT	0.00000	0.000136	0.00000	0.00000	na	0.06250	0	0	0	0	na	10
pair5_7	AC	0.06250	0.062364	0.06250	0.06250	na	0.00000	10	10	10	10	na	0

pair5_8	GC	0.91875	0.918750	0.91875	0.91875	na	na	147	147	147	147	na	na
pair5_8	GT	0.01875	0.018750	0.01875	0.01875	na	na	3	3	3	3	na	na
pair5_8	AC	0.06250	0.062500	0.06250	0.06250	na	na	10	10	10	10	na	na
pair5_8	AT	0.00000	0.00000	0.00000	0.00000	na	na	0	0	0	0	na	na
pair5_9	GT	0.07500	0.073133	0.06664	na	0.06664	0.07500	12	12	11	na	11	12
pair5_9	GC	0.86250	0.864367	0.87086	na	0.87086	0.86250	138	138	139	na	139	138
pair5_9	AT	0.00000	0.001867	0.00836	na	0.00836	0.00000	0	0	1	na	1	0
pair5_9	AC	0.06250	0.060633	0.05414	na	0.05414	0.06250	10	10	9	na	9	10
pair6_7	GT	0.87500	0.875000	0.87500	0.87500	na	na	140	140	140	140	na	na
pair6_7	GC	0.11875	0.118750	0.11875	0.11875	na	na	19	19	19	19	na	na
pair6_7	AT	0.00625	0.006250	0.00625	0.00625	na	na	1	1	1	1	na	na
pair6_7	AC	0.00000	0.00000	0.00000	0.00000	na	na	0	0	0	0	na	na
pair6_8	GC	0.97500	0.975000	0.97500	0.97500	na	na	156	156	156	156	na	na
pair6_8	GT	0.01875	0.018750	0.01875	0.01875	na	na	3	3	3	3	na	na
pair6_8	AC	0.00625	0.006250	0.00625	0.00625	na	na	1	1	1	1	na	na
pair6_8	AT	0.00000	0.00000	0.00000	0.00000	na	na	0	0	0	0	na	na
pair6_9	GT	0.07500	0.075000	0.07500	na	0.07500	na	12	12	12	na	12	na
pair6_9	GC	0.91875	0.918750	0.91875	na	0.91875	na	147	147	147	na	147	na
pair6_9	AC	0.00625	0.006250	0.00625	na	0.00625	na	1	1	1	na	1	na
pair6_9	AT	0.00000	0.00000	0.00000	na	0.00000	na	0	0	0	na	0	na

pair7_8	TC	0.86250	0.862500	0.86250	0.86250	na	na	138	138	138	138	na	na
pair7_8	TT	0.01875	0.018750	0.01875	0.01875	na	na	3	3	3	3	na	na
pair7_8	CC	0.11875	0.118750	0.11875	0.11875	na	na	19	19	19	19	na	na
pair7_8	CT	0.00000	0.00000	0.00000	0.00000	na	na	0	0	0	0	na	na
pair7_9	TT	0.07500	0.072926	0.07306	na	0.07306	0.07500	12	12	12	na	12	12
pair7_9	TC	0.80625	0.808324	0.80819	na	0.80819	0.80625	129	129	129	na	129	129
pair7_9	CT	0.00000	0.002074	0.00194	na	0.00194	0.00000	0	0	0	na	0	0
pair7_9	CC	0.11875	0.116676	0.11681	na	0.11681	0.11875	19	19	19	na	19	19
pair8_9	CT	0.07500	0.075000	0.07500	na	0.07500	na	12	12	12	na	12	na
pair8_9	CC	0.90625	0.906250	0.90625	na	0.90625	na	145	145	145	na	145	na
pair8_9	TC	0.01875	0.018750	0.01875	na	0.01875	na	3	3	3	na	3	na
pair8_9	TT	0.00000	0.00000	0.00000	na	0.00000	na	0	0	0	na	0	na
