# Supplementary material for: Cubic exact solutions for the estimation of pairwise haplotype frequencies: implications for linkage disequilibrium analyses and a web tool 'CubeX'
Source: BMC Bioinformatics. 2007 Nov 2;8:428. doi: 10.1186/1471-2105-8-428 (PMC2180187; doi:10.1186/1471-2105-8-428)
Supplement: Additional file 2 — Comparisons of PHASE, MIDAS and CubeX on HapMap IGF2 region data (from , [23,24]). A comparison of PHASE, MIDAS and CubeX for pairwise analysis of genotype data derived from statistically inferred long-range multi-locus haplotypes. [file 1471-2105-8-428-S2.pdf]

Supplementary File 2: Comparisons of PHASE, MIDAS and CubeX (CubeX-a, CubeX-b and CubeX-g for three solutions, na where a solution is not biologically possible) on HapMap *IGF2* region data (www.hapmap.org)

pop: CEU, **build**: ncbi_b35, **hapmap_release**: rel2, **filters**: poly_CEU, **start**: 2093554, **stop**: 2133553,

snps: rs3802971, rs734351 , rs3213221, rs4244808, rs1003483, rs3741208, rs1004446, rs4320932 and rs7924316

		Frequencies						Counts					
**Pair**	**Haplotype**	**Real**	**PHASE**	**MIDAS**	**CubeX-a**	**CubeX-b**	**CubeX-g**	**Real**	**PHASE**	**MIDAS**	**CubeX-a**	**CubeX-b**	**CubeX-g**
pair1_2	AA	0	0	0	0	na	na	0	0	0	0	na	na
pair1_2	AG	0.07	0.07	0.07	0.07	na	na	8	8	8	8	na	na
pair1_2	GA	0.53	0.53	0.53	0.53	na	na	64	64	64	64	na	na
pair1_2	GG	0.4	0.4	0.4	0.4	na	na	48	48	48	48	na	na
pair1_3	AC	0.07	0.07	0.07	0.07	na	na	8	8	8	8	na	na
pair1_3	AG	0	0	0	0	na	na	0	0	0	0	na	na
pair1_3	GC	0.4	0.4	0.4	0.4	na	na	48	48	48	48	na	na
pair1_3	GG	0.53	0.53	0.53	0.53	na	na	64	64	64	64	na	na
pair1_4	AG	0.07	0.07	0.07	0.07	na	na	8	8	8	8	na	na
pair1_4	AT	0	0	0	0	na	na	0	0	0	0	na	na
pair1_4	GG	0.38	0.38	0.38	0.38	na	na	46	46	46	46	na	na
pair1_4	GT	0.55	0.55	0.55	0.55	na	na	66	66	66	66	na	na
pair1_5	AG	0.07	0.07	0.07	0.07	na	na	8	8	8	8	na	na
pair1_5	AT	0	0	0	0	na	na	0	0	0	0	na	na

pair1_5	GG	0.41	0.41	0.41	0.41	na	na	49	49	49	49	na	na
pair1_5	GT	0.53	0.52	0.53	0.53	na	na	63	63	63	63	na	na
pair1_6	AA	0	0	0	0	na	na	0	0	0	0	na	na
pair1_6	AG	0.07	0.07	0.07	0.07	na	na	8	8	8	8	na	na
pair1_6	GA	0.36	0.36	0.36	0.36	na	na	43	43	43	43	na	na
pair1_6	GG	0.58	0.58	0.58	0.58	na	na	69	69	69	69	na	na
pair1_7	AA	0.07	0.07	0.07	0.07	na	na	8	8	8	8	na	na
pair1_7	AG	0	0	0	0	na	na	0	0	0	0	na	na
pair1_7	GA	0.34	0.34	0.34	0.34	na	na	41	41	41	41	na	na
pair1_7	GG	0.59	0.59	0.59	0.59	na	na	71	71	71	71	na	na
pair1_8	AC	0	0	0	0	na	na	0	0	0	0	na	na
pair1_8	AT	0.06	0.07	0.07	0.07	na	na	7	8	8	8	na	na
pair1_8	GC	0.17	0.2	0.2	0.2	na	na	20	24	24	24	na	na
pair1_8	GT	0.61	0.73	0.73	0.73	na	na	73	88	88	88	na	na
pair1_9	AG	0.06	0.06	0.06	0.06	na	na	7	7	7	7	na	na
pair1_9	AT	0.01	0.01	0.01	0.01	na	na	1	1	1	1	na	na
pair1_9	GG	0.48	0.49	0.48	0.48	na	na	58	58	58	58	na	na
pair1_9	GT	0.45	0.45	0.45	0.45	na	na	54	54	54	54	na	na
pair2_3	AC	0	0	0	0	na	na	0	0	0	0	na	na

pair2_3	AG	0.53	0.53	0.53	0.53	na	na	64	64	64	64	na	na
pair2_3	GC	0.47	0.47	0.47	0.47	na	na	56	56	56	56	na	na
pair2_3	GG	0	0	0	0	na	na	0	0	0	0	na	na
pair2_4	AG	0.35	0.36	0.36	0.36	na	na	42	43	43	43	na	na
pair2_4	AT	0.18	0.17	0.17	0.17	na	na	22	21	21	21	na	na
pair2_4	GG	0.1	0.09	0.09	0.09	na	na	12	11	11	11	na	na
pair2_4	GT	0.37	0.38	0.38	0.38	na	na	44	45	45	45	na	na
pair2_5	AG	0.37	0.39	0.39	0.39	na	na	44	46	47	47	na	na
pair2_5	AT	0.17	0.15	0.15	0.15	na	na	20	18	17	17	na	na
pair2_5	GG	0.11	0.09	0.09	0.09	na	na	13	11	10	10	na	na
pair2_5	GT	0.36	0.38	0.38	0.38	na	na	43	45	46	46	na	na
pair2_6	AA	0.35	0.35	0.35	0.35	na	na	42	42	42	42	na	na
pair2_6	AG	0.18	0.18	0.18	0.18	na	na	22	22	22	22	na	na
pair2_6	GA	0.01	0.01	0.01	0.01	na	na	1	1	1	1	na	na
pair2_6	GG	0.46	0.46	0.46	0.46	na	na	55	55	55	55	na	na
pair2_7	AA	0.16	0.12	0.12	0.12	na	na	19	15	14	14	na	na
pair2_7	AG	0.38	0.41	0.42	0.42	na	na	45	49	50	50	na	na
pair2_7	GA	0.25	0.29	0.29	0.29	na	na	30	34	35	35	na	na
pair2_7	GG	0.22	0.18	0.18	0.18	na	na	26	22	21	21	na	na

pair2_8	AC	0	0	0	0	na	na	0	0	0	0	na	na
pair2_8	AT	0.53	0.53	0.53	0.53	na	na	64	64	64	64	na	na
pair2_8	GC	0.2	0.2	0.2	0.2	na	na	24	24	24	24	na	na
pair2_8	GT	0.27	0.27	0.27	0.27	na	na	32	32	32	32	na	na
pair2_9	AG	0.42	0.41	0.41	0.41	na	na	50	49	49	49	na	na
pair2_9	AT	0.12	0.13	0.12	0.12	na	na	14	15	15	15	na	na
pair2_9	GG	0.13	0.14	0.13	0.13	na	na	15	16	16	16	na	na
pair2_9	GT	0.34	0.33	0.33	0.33	na	na	41	40	40	40	na	na
pair3_4	CG	0.1	0.09	0.09	0.09	na	na	12	11	11	11	na	na
pair3_4	CT	0.37	0.38	0.38	0.38	na	na	44	45	45	45	na	na
pair3_4	GG	0.35	0.36	0.36	0.36	na	na	42	43	43	43	na	na
pair3_4	GT	0.18	0.17	0.17	0.17	na	na	22	21	21	21	na	na
pair3_5	CG	0.11	0.09	0.09	0.09	na	na	13	11	10	10	na	na
pair3_5	CT	0.36	0.38	0.38	0.38	na	na	43	45	46	46	na	na
pair3_5	GG	0.37	0.39	0.39	0.39	na	na	44	46	47	47	na	na
pair3_5	GT	0.17	0.15	0.15	0.15	na	na	20	18	17	17	na	na
pair3_6	CA	0.01	0.01	0.01	0.01	na	na	1	1	1	1	na	na
pair3_6	CG	0.46	0.46	0.46	0.46	na	na	55	55	55	55	na	na
pair3_6	GA	0.35	0.35	0.35	0.35	na	na	42	42	42	42	na	na
pair3_6	GG	0.18	0.18	0.18	0.18	na	na	22	22	22	22	na	na

pair3_7	CA	0.25	0.29	0.29	0.29	na	na	30	34	35	35	na	na
pair3_7	CG	0.22	0.18	0.18	0.18	na	na	26	22	21	21	na	na
pair3_7	GA	0.16	0.12	0.12	0.12	na	na	19	15	14	14	na	na
pair3_7	GG	0.38	0.41	0.42	0.42	na	na	45	49	50	50	na	na
pair3_8	CC	0.2	0.2	0.2	0.2	na	na	24	24	24	24	na	na
pair3_8	CT	0.27	0.27	0.27	0.27	na	na	32	32	32	32	na	na
pair3_8	GC	0	0	0	0	na	na	0	0	0	0	na	na
pair3_8	GT	0.53	0.53	0.53	0.53	na	na	64	64	64	64	na	na
pair3_9	CG	0.13	0.14	0.13	0.13	na	na	15	16	16	16	na	na
pair3_9	CT	0.34	0.33	0.33	0.33	na	na	41	40	40	40	na	na
pair3_9	GG	0.42	0.41	0.41	0.41	na	na	50	49	49	49	na	na
pair3_9	GT	0.12	0.13	0.12	0.12	na	na	14	15	15	15	na	na
pair4_5	GG	0.45	0.45	0.45	0.45	na	na	54	54	54	54	na	na
pair4_5	GT	0	0	0	0	na	na	0	0	0	0	na	na
pair4_5	TG	0.03	0.03	0.03	0.03	na	na	3	3	3	3	na	na
pair4_5	TT	0.53	0.52	0.53	0.53	na	na	63	63	63	63	na	na
pair4_6	GA	0.33	0.34	0.34	0.34	na	na	40	41	41	41	na	na
pair4_6	GG	0.12	0.11	0.11	0.11	na	na	14	13	13	13	na	na
pair4_6	TA	0.03	0.02	0.02	0.02	na	na	3	2	2	2	na	na

pair4_6	TG	0.53	0.53	0.53	0.53	na	na	63	64	64	64	na	na
pair4_7	GA	0.12	0.12	0.11	0.11	na	na	14	14	13	13	na	na
pair4_7	GG	0.33	0.33	0.34	0.34	na	na	40	40	41	41	na	na
pair4_7	TA	0.29	0.29	0.3	0.3	na	na	35	35	36	36	na	na
pair4_7	TG	0.26	0.26	0.25	0.25	na	na	31	31	30	30	na	na
pair4_8	GC	0	0	0	0	na	na	0	0	0	0	na	na
pair4_8	GT	0.45	0.45	0.45	0.45	na	na	54	54	54	54	na	na
pair4_8	TC	0.2	0.2	0.2	0.2	na	na	24	24	24	24	na	na
pair4_8	TT	0.35	0.35	0.35	0.35	na	na	42	42	42	42	na	na
pair4_9	GG	0.43	0.43	0.43	0.43	na	na	52	52	52	52	na	na
pair4_9	GT	0.02	0.02	0.02	0.02	na	na	2	2	2	2	na	na
pair4_9	TG	0.11	0.11	0.11	0.11	na	na	13	13	13	13	na	na
pair4_9	TT	0.44	0.44	0.44	0.44	na	na	53	53	53	53	na	na
pair5_6	GA	0.34	0.34	0.34	0.34	na	na	41	41	41	41	na	na
pair5_6	GG	0.13	0.14	0.14	0.14	na	na	16	16	16	16	na	na
pair5_6	TA	0.02	0.02	0.02	0.02	na	na	2	2	2	2	na	na
pair5_6	TG	0.51	0.51	0.51	0.51	na	na	61	61	61	61	na	na
pair5_7	GA	0.13	0.13	0.12	0.12	na	na	15	15	14	14	na	na
pair5_7	GG	0.35	0.35	0.36	0.36	na	na	42	42	43	43	na	na

pair5_7	TA	0.28	0.28	0.29	0.29	na	na	34	34	35	35	na	na
pair5_7	TG	0.24	0.24	0.24	0.24	na	na	29	29	28	28	na	na
pair5_8	GC	0.01	0	0	0	na	na	1	0	0	0	na	na
pair5_8	GT	0.47	0.47	0.48	0.48	na	na	56	57	57	57	na	na
pair5_8	TC	0.19	0.2	0.2	0.2	na	na	23	24	24	24	na	na
pair5_8	TT	0.33	0.33	0.33	0.33	na	na	40	39	39	39	na	na
pair5_9	GG	0.45	0.46	0.46	0.46	na	na	54	55	55	55	na	na
pair5_9	GT	0.03	0.02	0.02	0.02	na	na	3	2	2	2	na	na
pair5_9	TG	0.09	0.09	0.08	0.08	na	na	11	10	10	10	na	na
pair5_9	TT	0.43	0.44	0.44	0.44	na	na	52	53	53	53	na	na
pair6_7	AA	0	0	0	0	na	na	0	0	0	0	na	na
pair6_7	AG	0.36	0.36	0.36	0.36	na	na	43	43	43	43	na	na
pair6_7	GA	0.41	0.41	0.41	0.41	na	na	49	49	49	49	na	na
pair6_7	GG	0.23	0.23	0.23	0.23	na	na	28	28	28	28	na	na
pair6_8	AC	0	0	0	0	na	na	0	0	0	0	na	na
pair6_8	AT	0.36	0.36	0.36	0.36	na	na	43	43	43	43	na	na
pair6_8	GC	0.2	0.2	0.2	0.2	na	na	24	24	24	24	na	na
pair6_8	GT	0.44	0.44	0.44	0.44	na	na	53	53	53	53	na	na
pair6_9	AG	0.33	0.33	0.33	0.33	na	na	40	39	39	39	na	na

pair6_9	AT	0.03	0.03	0.03	0.03	na	na	3	4	4	4	na	na
pair6_9	GG	0.21	0.22	0.21	0.21	na	na	25	26	26	26	na	na
pair6_9	GT	0.43	0.43	0.43	0.43	na	na	52	51	51	51	na	na
pair7_8	AC	0	0	0	0	na	na	0	0	0	0	na	na
pair7_8	AT	0.41	0.41	0.41	0.41	na	na	49	49	49	49	na	na
pair7_8	GC	0.2	0.2	0.2	0.2	na	na	24	24	24	24	na	na
pair7_8	GT	0.39	0.39	0.39	0.39	na	na	47	47	47	47	na	na
pair7_9	AG	0.18	0.16	0.15	0.15	na	na	22	19	18	18	na	na
pair7_9	AT	0.23	0.25	0.26	0.26	na	na	27	30	31	31	na	na
pair7_9	GG	0.36	0.38	0.39	0.39	na	na	43	46	47	47	na	na
pair7_9	GT	0.23	0.21	0.2	0.2	na	na	28	25	24	24	na	na
pair8_9	CG	0	0	0	0	na	na	0	0	0	0	na	na
pair8_9	CT	0.2	0.2	0.2	0.2	na	na	24	24	24	24	na	na
pair8_9	TG	0.54	0.54	0.54	0.54	na	na	65	65	65	65	na	na
pair8_9	TT	0.26	0.26	0.26	0.26	na	na	31	31	31	31	na	na
